# GSK3β as a potential regulator in AML: A pan-cancer multi-omics analysis

**DOI:** 10.1371/journal.pone.0344994

**Published:** 2026-03-31

**Authors:** Ruyi Qiu, Yingying Zhou, Shuang Song, Zhejiong Wang

**Affiliations:** 1 Department of Laboratory Medicine, The First Affiliated Hospital of Zhejiang Chinese Medical University (Zhejiang Provincial Hospital of Chinese Medicine), Hangzhou, Zhejiang Province, China; 2 Department of Laboratory Medicine, Huangyan Hospital of Wenzhou Medical University, Taizhou First People’s Hospital, Taizhou, Zhejiang Province, China; 3 Department of Pathology, Zhejiang Provincial Hospital of Traditional Chinese Medicine, Hangzhou, Zhejiang Province, China; Xiangya Hospital Central South University, CHINA

## Abstract

The distinct roles of GSK3 isoforms (GSK3α/β) in tumorigenesis and immune modulation remain poorly characterized across malignancies. We integrated multi-omics data from TCGA, GTEx, and single-cell RNA-seq to analyze GSK3α/β expression patterns in 31 cancers. Functional clustering, survival analysis (Cox regression), immune infiltration, and in vitro validation (AML cell lines treated with CHIR-99021) were performed. Integrated multi-omics analysis of 31 malignancies revealed divergent dysregulation of GSK3 isoforms: GSK3α was upregulated in 19 solid tumors but suppressed in AML, while GSK3β was elevated in 23 cancers and high-risk AML subtypes (FAB-M0/M1, *P* = 0.0013). GSK3β outperformed GSK3α as a pan-cancer diagnostic biomarker, achieving superior AUC in 9 tumors. Prognostically, high GSK3α predicted poor OS in ACC (HR = 8.80, *P* = 0.0047) and MESO (HR = 2.75, *P* < 0.0067), whereas GSK3β independently stratified cytogenetic-risk AML (HR = 4.22, *P* = 0.007). Immune profiling uncovered isoform-specific TME modulation: GSK3α correlated with protumorigenic immune infiltration (Treg/Th17, r = 0.38), contrasting GSK3β’s broad negative associations with cytotoxic effectors (r = −0.27). Functional validation in AML THP-1 cells demonstrated that the GSK3 inhibitor CHIR-99021 (10 μM) significantly suppressed proliferation, induced apoptosis, and caused S-phase cell cycle arrest, concomitant with downregulation of c-Myc. These findings establish GSK3β as a key regulator of oncogenic programs in AML. This study provides a comprehensive pan-cancer atlas of GSK3 isoform-specific functionality, nominating GSK3β as a high-priority therapeutic target.

## Introduction

Cancer persists as a leading global cause of mortality with profound socioeconomic burdens. Current therapeutic modalities face constraints: immune checkpoint inhibitors such as PD-1/PD-L1 blockers achieved durable responses in only 15–20% of solid tumors due to primary resistance and immune-related adverse events [1–3]; targeted agents exhibited response rates below 40% in KRAS-mutant cancers [4]; and chemotherapy induced polyploid giant cancer cells through miR-1246/GSK3β/β-catenin axis activation, driving chemoresistance in NSCLC [5]. Acute myeloid leukemia (AML) exemplified these challenges with a 5-year survival rate of 25% and frequent therapeutic failures [6–8].

Glycogen synthase kinase 3 (GSK3), a multifunctional kinase integrating insulin/IGF, Wnt/β-catenin, Hedgehog, NF-κB, and TGF-β pathways [9–12], demonstrates context-dependent duality in oncogenesis. It promoted tumor progression via miR-1246-mediated GSK3β inactivation in NSCLC (enhancing β-catenin signaling and PGCC-associated chemoresistance) [5] and HIF1α stabilization in breast cancer (accelerating stemness through LINC00115/SETDB1) [13], yet suppresses AKT-driven tumorigenesis in RCC by phosphorylating PRR11 for FBXW7-dependent ubiquitination [14]. Although GSK3 inhibitors showed promise—Combination therapy of LY2090314 and nab-paclitaxel significantly prolongs median survival in orthotopic pancreatic cancer xenograft nude mouse models [15]. And covalent inhibitors overcome ATRA resistance in leukemia [16]—persistent barriers include isoform-specific functional antagonism (e.g., GSK3α-enhanced immunity vs. GSK3β-suppressed cytotoxicity in AML) [17–19] and uncharacterized pan-cancer expression patterns with diagnostic/prognostic correlations. Emerging evidence has demonstrated the critical role of Glycogen Synthase Kinase-3 (GSK-3) in modulating anti-tumor immune responses. In recent years, numerous GSK-3 inhibitors have been developed, with several advances to clinical trials. However, pan-cancer expression patterns of GSK-3 and their diagnostic/prognostic relevance remain largely unexplored.

To address these gaps, we performed integrated multi-omics analyses across 31 malignancies combined with immune microenvironment profiling and AML cellular validation, aiming to establish pan-cancer landscapes of GSK3α/β expression and clinical associations, elucidate isoform-specific immunomodulatory mechanisms, and provide novel insights into precision oncology paradigms.

## Materials and methods

### 1. Analysis of GSK3α/β expression

Bioinformatics analyses were conducted using the Xiantao Academic online tool (https://www.xiantao.love/), which employs standard R packages for computation. Transcriptomic datasets for subgroup comparison were acquired from the UCSC XENA platform (https://xenabrowser.net/datapages), encompassing uniformly processed Toil pipeline [20] RNA-seq data in GSK3α/β formats from both TCGA (tumor samples: n = 9,807; adjacent normal tissues: n = 727) and GTEx (normal tissues: n = 7,568). The pan-cancer analysis involved log2(value+1) transformation of expression values. Statistical comparisons between tumor and normal groups were performed using Wilcoxon rank-sum tests, with detailed test statistics and degrees of freedom documented in supplementary materials.

For paired sample analysis, matched adjacent normal tissues -cancerous datasets in GSK3α/β format were directly retrieved from the TCGA portal (https://portal.gdc.cancer.gov). Intragroup comparisons were conducted using Wilcoxon signed-rank tests, the complete statistical outputs of which were provided in supplementary materials.

Normal hematopoiesis and chronic myeloid leukemia (CML) specimens were retrieved from the GSE4170 (Platform: GPL2029 Rosetta/Merck Human 25k v2.2.1 microarray). For acute myeloid leukemia (AML) cohorts, expression matrices from UCSC XENA (https://xenabrowser.net/datapages/) were processed, including 173 AML cases with matched normal controls. To clarify the role of GSK3α/β in leukemia stratification, RNA-seq data derived from TCGA (https://portal.gdc.cancer.gov) were retrieved. This was related to bone marrow blast percentages and the French-American-British (FAB) classification systems, establishing quantitative associations between kinase expression and disease subtyping. The RNA-seq data (in transcripts per million, TPM, format) and the comprehensive clinical metadata for the TCGA-LAML cohort were obtained from the GDC Data Portal (https://portal.gdc.cancer.gov) using the TCGAbiolinks R package. Gene expression values were log2-transformed after adding a pseudo-count of 1. Clinical variables, including the French-American-British (FAB) classification and cytogenetic risk category, were extracted directly from the clinical metadata file. To ensure a clean cohort for clinical association studies, the following sequential filters were applied: 1) removal of non-tumor samples, 2) exclusion of samples with missing (NA or blank) entries for either FAB or cytogenetic risk variables, and 3) retention of only the first entry for any patient with potential duplicate samples. The final sample counts (N) after this curation process are specified in the respective results. All statistical analyses involving these clinical variables were performed in R (v4.2.1) with core functions from the stats package and auxiliary functions from the car (v3.1-0) and ggplot2 (v3.3.6) packages.

### 2. Analysis of the prognostic and diagnostic value of GSK3α/β

The prognostic significance of GSK3α/β expression across malignancies was investigated through survival analysis conducted. Tumor cohorts were stratified into low and high GSK3α/β expression subgroups using quartile-based cutoff values (25th and 75th percentiles) for subsequent comparative survival analysis. Univariate Cox proportional hazards models were fitted using the survival R package (v3.3-1) to calculate hazard ratios and P-values. The proportional hazards assumption was tested for significant models using the cox.zph function.

To evaluate the diagnostic potential of GSK3α/β, receiver operating characteristic curve analysis was performed. Primary tumor samples were defined as the “positive” class, and the corresponding adjacent normal tissue samples from TCGA or normal tissue from GTEx were defined as the “negative” class. The area under the curve (AUC) and its 95% confidence interval (calculated via DeLong’s test) were computed using the pROC package (v1.18.0) to quantify and compare diagnostic performance. Diagnostic performance was categorized as follows: limited discriminative power (AUC 0.5–0.7), moderate accuracy (AUC 0.7–0.9), and high predictive validity (AUC > 0.9). The pan-cancer survival and ROC analyses in this study are exploratory and screening in nature. Consequently, P-values from the survival analysis are reported as nominal values without multiple testing adjustment, and the ROC analysis focuses on reporting AUC values with DeLong confidence intervals for cross-marker comparison. We note that more advanced internal validation methods, such as bootstrap resampling, could be applied in future studies for a more in-depth assessment of specific candidate biomarkers identified here.

### 3. Immune microenvironment profiling and correlation analysis of GSK3α/β

The enrichment scores of 24 immune cell subtypes within the tumor microenvironment were quantified using the single-sample Gene Set Enrichment Analysis (ssGSEA) algorithm. This analysis was implemented through the GSVA R package (version 1.46.0), utilizing the well-established gene signatures for 24 immune cell types published by Bindea [21,22]. To evaluate the association between GSK3α/β expression and immune infiltration patterns across malignancies, Spearman’s rank correlation coefficients were computed. To account for multiple hypothesis testing arising from the analysis of 24 cell types across multiple cancers, the resulting P-values were adjusted using the Benjamini-Hochberg false discovery rate (FDR) method. Significant correlations are reported at an FDR-adjusted P-value (i.e., q-value) < 0.05. The correlation matrices were visualized as hierarchical clustering heatmaps using the pheatmap package.

### 4. Enrichment and function analysis

RNA sequencing data from the TCGA-LAML cohort were retrieved from the TCGA portal and preprocessed to isolate GSK3α/β-associated expression profiles. Samples were stratified into high- and low-expression cohorts based on a quartile cutoff of GSK3α/β transcript levels. Differentially expressed genes (DEGs) between the two groups were identified through comparative analysis with a significance threshold of |log2 fold change| > 1 and adjusted p-value < 0.05. Functional annotation and pathway enrichment analyses were subsequently conducted via Gene Ontology (GO) and Kyoto Encyclopedia of Genes and Genomes (KEGG) databases, implemented using the “ClusterProfiler” R package (v4.0).

### 5. Cell Culture and Treatment

THP-1 human leukemia cells were obtained from the National Collection of Authenticated Cell Cultures (Shanghai, China) and cultured in RPMI-1640 medium (Sigma-Aldrich, R7256, USA) containing 10% fetal bovine serum (Serana, S-FBS-SA-015, Germany) and 1% penicillin/streptomycin (Gibco, Thermo Fisher Scientific, 15140122, Waltham, MA, USA). Cells were maintained at 37°C in a humidified 5% CO₂ incubator. For GSK3 inhibition, cells were treated with the selective inhibitor CHIR-99021 (Selleck, S1263, Houston, Texas, USA) prior to functional assays for 48-hour exposure.

### 6. Apoptosis assay, cell cycle assay

Cell cycle analysis: Following 48-hour treatment with 10 µM CHIR-99021 or DMSO control in 24-well plates, approximately 1 × 10⁶ THP-1 cells were collected by centrifugation (1000 rpm, 5 min). The cell pellet was washed once with PBS and resuspended in 1 mL of DNA Staining Solution (MultiSciences, CCS012, China) containing 10 µL of Permeabilization Solution. After vortexing for 5–10 seconds, the cells were incubated at room temperature in the dark for 30 minutes. Cell cycle distribution was analyzed immediately by flow cytometry.

Apoptosis assay: THP-1 cells were treated and collected as described above. The cell pellet was resuspended and washed once with 1 mL of ice-cold PBS. After centrifugation, cells were resuspended in 1X Binding Buffer (prepared by 1:9 dilution of the stock solution with deionized water) at a concentration of 1–5 × 10⁶ cells/mL. A 100 µL aliquot of the cell suspension was incubated with 5 µL of Annexin V-FITC (Share Bio, CA1020, China) for 5 minutes at room temperature in the dark. Subsequently, 5 µL of propidium iodide (PI) was added, followed by 400 µL of PBS. The samples were analyzed by Beckman Coulter DxFLEX flow cytometer (Jiangsu, China) immediately.

### 7. Cell proliferation assay

Cell growth kinetics were evaluated via CCK-8 colorimetric assay (Beyotime, C0038, China). THP-1 cells were seeded in 96-well plates at a density of 4 × 10³ cells/well in 100 µL of complete medium. After adherence (or directly for suspension cells), cells were treated with 10 µM CHIR-99021 or an equal volume of DMSO (vehicle control) for 48 hours. CCK-8 reagent (10 µL per well) was then added, followed by incubation for 2 hours at 37°C. Absorbance at 450 nm was measured using a BioTek Synergy H1 microplate reader. Viability data were expressed as relative viability normalized to untreated controls.

### 8. Flow cytometry

All flow cytometry samples were acquired on a Beckman Coulter DxFLEX flow cytometer using CytExpert software (v2.4). A standardized gating strategy was applied: cells were first gated on a forward scatter area vs. side scatter area (FSC-A/SSC-A) plot to exclude debris, followed by singlet selection using a forward scatter height vs. area (FSC-H/FSC-A) plot. Fluorescence-minus-one (FMO) controls were used in all relevant experiments to determine background fluorescence and set appropriate gates. For apoptosis analysis, fluorescence was measured in the FITC (Annexin V) and ECD (PI) channels. Data were analyzed using CytExpert software (Beckman Coulter, version 2.4). Quadrant statistics were established based on unstained cells and single-stained controls to distinguish viable (Annexin V ⁻ /PI⁻), early apoptotic (Annexin V ⁺ /PI⁻), late apoptotic (Annexin V ⁺ /PI⁺), and necrotic (Annexin V ⁻ /PI⁺) cell populations. For cell cycle analysis, the FL3-A channel was used to detect PI fluorescence. A minimum of 10,000 singlet events, gated based on FSC-H vs. FSC-A, were collected per sample at the lowest acquisition speed. The resulting DNA content histograms were analyzed using ModFit LT software (Verity Software House, version 5.0) to determine the percentage of cells in G0/G1, S, and G2/M phases.

### 9. Western blot analysis

Western blot analysis was performed according to established methodologies [23]. Membranes were incubated with the following primary antibodies: anti-GSK-3β(Proteintech, 22104–1-AP, Wuhan, China)a, anti-c-Myc(Proteintech, 10828–1-AP, Wuhan, China), PAPR1(Proteintech, 66520–1-Ig, Wuhan, China)and anti-Tubulin (Proteintech, 80762–1-RR, Wuhan, China); anti-p-GSK3β(Cell Signaling Technology, 5558T, Danvers, MA, USA), anti-CCND1 (Cell Signaling Technology, 55506S, Danvers, MA, USA). Following three TBST washes, protein bands were detected using HRP-conjugated secondary antibodies (Beyotime, A0216 and A0208, China) through 1-hour room temperature incubation.

### 10. Statistical analysis

All functional experiments were performed with n = 3 independent biological replicates, each with 2–3 technical replicates. The experimental results were expressed as mean ± SD and analyzed by using GraphPad Prism software (version 8.0; San Diego, CA, USA). For bioinformatics analyses comparing molecules across multiple groups, the Wilcoxon rank sum test was applied, while experimental data from cellular models were analyzed using the two-tailed unpaired Student’s t-test. All p-values reported in this study were calculated via two-tailed tests, with a threshold of *p* < 0.05 defined as statistically significant differences.

## Results

### GSK3α/β expression profiles across malignancies and its functional clustering

**Fig 1:**
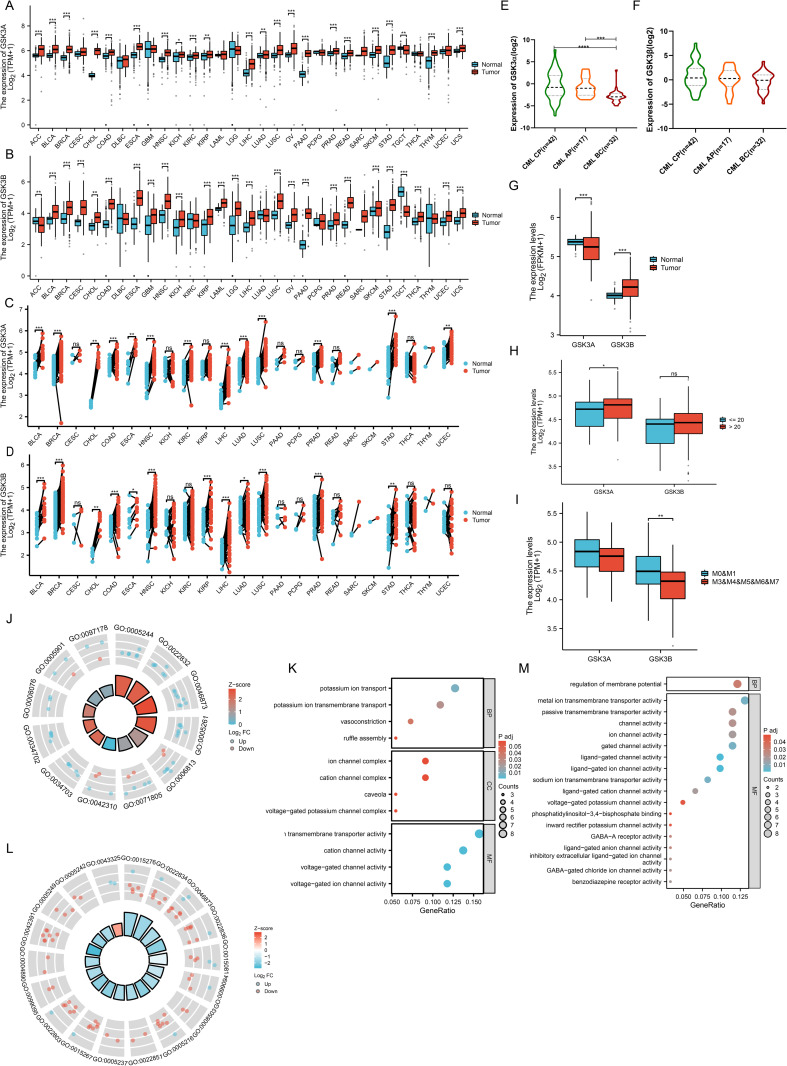
Pan-cancer landscape of GSK3 isoform expression and functional clustering. (A-B) Expression of GSK3α and GSK3β across 31 malignancies from TCGA-GTEx integration (N = 17,936). (C-D) Pairwise comparison (N = 1,404) of GSK3α and GSK3β from the TCGA database. (E-G) Hematologic malignancy: (E) GSK3α suppression during CML progression from GEO database (CP: N = 42; AP: N = 17; BC: N = 32); (F) GSK3β remained unchanged during the progression of CML; (G) GSK3α suppression while GSK3β elevation in AML. (H-I) AML stratification: (H) GSK3α association with BM-blast burden; (I) GSK3β enrichment in FAB-M0/M1 subtypes. (J-M) Functional clustering: (J) (L) Enrichment circle diagram of differential gene expression in acute leukemia by GO/KEGG enrichment analysis. Patients were divided into high (top 25%) and low (bottom 25%) GSK3-expression (GSK3α and GSK3β) groups. J: GSK3α; L: GSK3β. (K) (M) The biological functions of AML were analyzed based on the levels of GSK3α and GSK3β through GO and KEGG analyses. Patients were divided into high (top 25%) and low (bottom 25%) GSK3-expression (GSK3α and GSK3β) groups. K: GSK3α; M: GSK3β. Error bars, ± SD. *p < 0.05, **p < 0.01, ***p < 0.001.

Integrated analysis of TCGA and GTEx datasets demonstrated that GSK3α was significantly upregulated in 19 malignancies (including ACC, BLCA, BRCA, CHOL, COAD, ESCA, HNSC, KICH, KIRC, KIRP, LIHC, LUAD, LUSC, OV, PAAD, PRAD, READ, SKCM, STAD, THYM, UCS) but downregulated in TGCT (Fig 1A). However, GSK3β exhibited marked upregulation in 23 cancer types (BLCA, BRCA, CESC, CHOL, COAD, ESCA, GBM, HNSC, KICH, KIRP, LAML, LGG, LIHC, LUSC, OV, PAAD, PRAD, READ, SKCM, STAD, THCA, UCEC, UCS), while being downregulated in ACC and TGCT (Fig 1B). Notably, ACC exhibited divergent expression patterns between GSK3α and GSK3β. To validate these findings, we analyzed TCGA-matched tumor-normal paired samples, confirming persistent overexpression of GSK3α in 13 cancers (BLCA, BRCA, CHOL, COAD, ESCA, HNSC, KIRC, LIHC, LUAD, LUSC, PRAD, STAD, UCEC) and consistent upregulation of GSK3β in 12 malignancies (BLCA, BRCA, CHOL, COAD, ESCA, HNSC, KIRP, LIHC, LUAD, LUSC, PRAD, STAD) (Fig 1C-1D). In hematologic malignancies, GSK3α expression progressively decreased with CML progression (P = 0.0002; P = 0.0001) and was suppressed in AML (Fig 1E and 1G). Conversely, GSK3β showed significant upregulation in AML but no correlation with CML progression (P = 0.058) (Fig 1F-1G). Stratification analysis based on bone marrow blast (BM-blast) proportions revealed elevated GSK3α expression in the high-blast cohort (> 20%, P = 0.047), whereas GSK3β exhibited preferential expression in FAB-M0/M1 AML subtypes (P = 0.0013) (Fig 1H-1I).

The cohort was stratified by GSK3α/β expression levels using quartile partitioning, with the upper and lower quartiles defining high- and low-expression cohorts respectively. Comparative transcriptomic profiling identified differentially expressed genes (DEGs), followed by functional annotation through Gene Ontology and KEGG pathway analyses to elucidate their biological significance. Analytical findings revealed partial functional overlap between GSK3α and GSK3β in ion channel regulation and ion transport processes (Fig 1J-1K). Notably, GSK3β exhibited broader biological involvement, including phosphatidylinositol-3,4-bisphosphate binding, γ-aminobutyric acid (GABA) receptor activity, and benzodiazepine receptor activity (Fig 1L-1M).

### Correlation of GSK3α/β with the diagnosis of pan-cancer and AML phenotype

**Fig 2:**
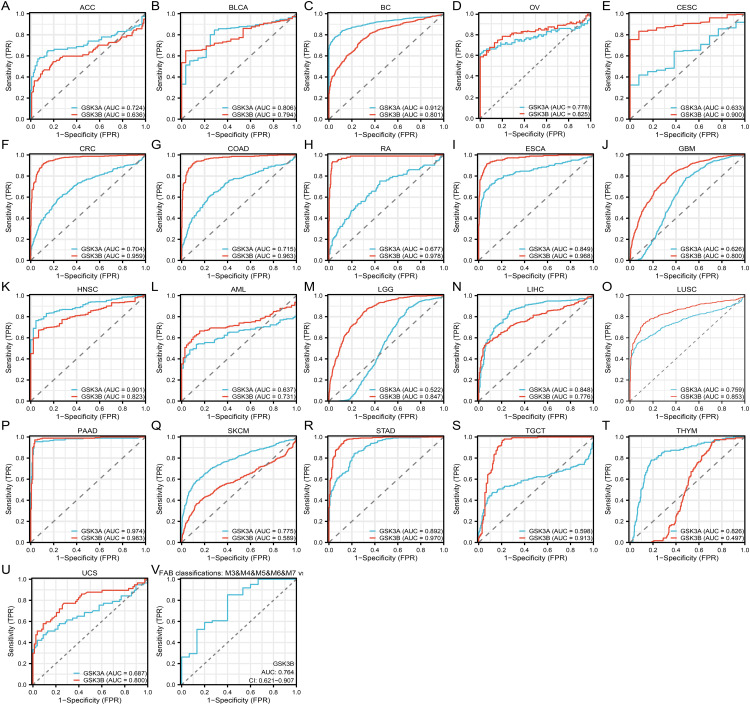
Diagnostic value of GSK3 isoforms across malignancies and AML subtypes. (A-U) ROC curves of GSK3α and GSK3β for pan-cancer diagnostic performance: (A) ACC (N = 77); (B) BLCA (N = 407); (C) BC (N = 1099); (D) OV (N = 427); (E) CESC (N = 306); (F) CRC (N = 383); (G) COAD (N = 290); (H) RA (N = 93); (I) ESCA (N = 182); (J) GBM (N = 689); (K) HNSC (N = 502); (L) AML (N = 173); (M) LGG (N = 523); (N) LIHC (N = 371); (O) LUSC (N = 498); (P) PAAD (N = 179); (Q) SKCM (N = 469); (R) STAD (N = 414); (S) TGCT (N = 154); (T) THYM (N = 119); (U) UCS (N = 57). (V) GSK3β predictive capacity for FAB-M0 AML (M0, N = 15; M3&M4&M5&M6&M7, N = 61). *p < 0.05, **p < 0.01, ***p < 0.001.

Receiver operating characteristic (ROC) analysis was employed to systematically evaluate the pan-cancer diagnostic potential of GSK3α/β isoforms. GSK3α demonstrated limited diagnostic capacity in CESC (AUC = 0.633) (Fig 2E), READ (AUC = 0.677) (Fig 2H), GBM (AUC = 0.626) (Fig 2J), AML (AUC = 0.637) (Fig 2L), and UCS (AUC = 0.687) (Fig 2U). Moderate predictive accuracy was observed for ACC (AUC = 0.724) (Fig. 2A), BLCA (AUC = 0.806) (Fig. 2B), OV (AUC = 0.778) (Fig 2D), CRC (AUC = 0.704) (Fig 2F), COAD (AUC = 0.715) (Fig 2G), ESCA (AUC = 0.849) (Fig 2I), LIHC (AUC = 0.848) (Fig 2N), LUSC (AUC = 0.759) (Fig 2O), SKCM (AUC = 0.775) (Fig 2Q), STAD (AUC = 0.892) (Fig 2R) and THYM (AUC = 0.826) (Fig 2T). Notably, GSK3α exhibited superior diagnostic performance in BC (AUC = 0.912) (Fig 2C), HNSC (AUC = 0.901) (Fig 2K) and PAAD (AUC = 0.974) (Fig 2P). GSK3β showed restricted predictive value in ACC (AUC = 0.636) (Fig 2A), but achieved moderate diagnostic accuracy in BLCA (AUC = 0.794), BC (AUC = 0.801), OV (AUC = 0.825) (Fig 2B-D), GBM (AUC = 0.800), HNSC (0.823), AML (0.731), lower-grade glioma (LGG, AUC = 0.847), LIHC (AUC = 0.776), LUSC (AUC = 0.853), and UCS (AUC = 0.800) (Fig 2J-2O and 2U). Comparative analysis revealed the enhanced diagnostic efficacy of GSK3β in nine malignancies: CESC (AUC = 0.900), CRC (AUC = 0.959), COAD (AUC = 0.963), READ (AUC = 0.978), ESCA (AUC = 0.968) (Fig 2E-2I), PAAD (AUC = 0.983) (Fig 2P), STAD (AUC = 0.970) (Fig 2R), and testicular germ cell tumors (TGCT, AUC = 0.913) (Fig 2S).

Furthermore, we focused on the association between GSK3α/β expression and AML stratification. GSK3β demonstrated predictive capacity for FAB-M0 subtype classification (AUC = 0.764, 95% CI 0.621–0.907) (Fig 2V). Univariable logistic regression modeling with GSK3α/β as independent variables revealed distinct predictive capacities: Comparative analysis showed GSK3α moderately predicted bone marrow blast percentage >20% (OR = 2.64, 95% CI 1.34–5.20; p = 0.005), while exhibiting marginal significance in peripheral blood blast prediction (p = 0.073) (Table 1). Notably, GSK3β exhibited robust association with cytogenetic risk stratification (poor vs favorable: OR = 4.47, 95% CI 1.47–13.54; p = 0.008) (Table 2). The comprehensive profiling established GSK3α/β as pan-cancer diagnostic biomarkers, with GSK3β exhibiting significantly higher discriminative power across multiple tumor types.

**Table 1:** Association of GSK3α Expression with Clinical Subtypes in Acute Myeloid Leukemia.

Characteristics	Total (N)	OR (95% CI)	P value
Gender (Male vs. Female)	150	1.176 (0.617–2.240)	0.622
Age (> 60 vs. <= 60)	150	1.315 (0.687–2.520)	0.409
WBC count(x10^9/L) (> 20 vs. <= 20)	149	1.275 (0.670–2.426)	0.460
BM blasts (%) (> 20 vs. <= 20)	150	2.641 (1.341–5.200)	**0.005**
PB blasts (%) (> 70 vs. <= 70)	150	1.809 (0.946–3.458)	0.073
FAB classifications (M3&M4&M5&M6&M7 vs. M0)	76	0.847 (0.273–2.626)	0.773
Cytogenetic risk (Poor vs. Favorable)	66	1.462 (0.552–3.873)	0.445
Cytogenetics (+8&del(5)&del(7)&inv(16)&t(15;17)&t(8;21)&t(9;11) vs. Normal)	110	0.760 (0.350–1.653)	0.489

**Table 2:** Association of GSK3β Expression with Clinical Subtypes in Acute Myeloid Leukemia.

Characteristics	Total (N)	OR (95% CI)	P value
Gender (Male vs. Female)	150	0.850 (0.447–1.620)	0.622
Age (> 60 vs. <= 60)	150	0.609 (0.317–1.172)	0.138
WBC count(x10^9/L) (> 20 vs. <= 20)	149	1.028 (0.541–1.954)	0.933
BM blasts (%) (> 20 vs. <= 20)	150	1.183 (0.614–2.279)	0.616
PB blasts (%) (> 70 vs. <= 70)	150	0.765 (0.402–1.454)	0.414
FAB classifications (M3&M4&M5&M6&M7 vs. M0)	76	0.404 (0.127–1.281)	0.124
Cytogenetic risk (Poor vs. Favorable)	66	4.471 (1.476–13.543)	**0.008**
Cytogenetics (+8&del(5)&del(7)&inv(16)&t(15;17)&t(8;21)&t(9;11) vs. Normal)	110	0.613 (0.281–1.338)	0.219

### Correlation of GSK3α/β with the prognosis of pan-cancer and AML phenotype

**Fig 3:**
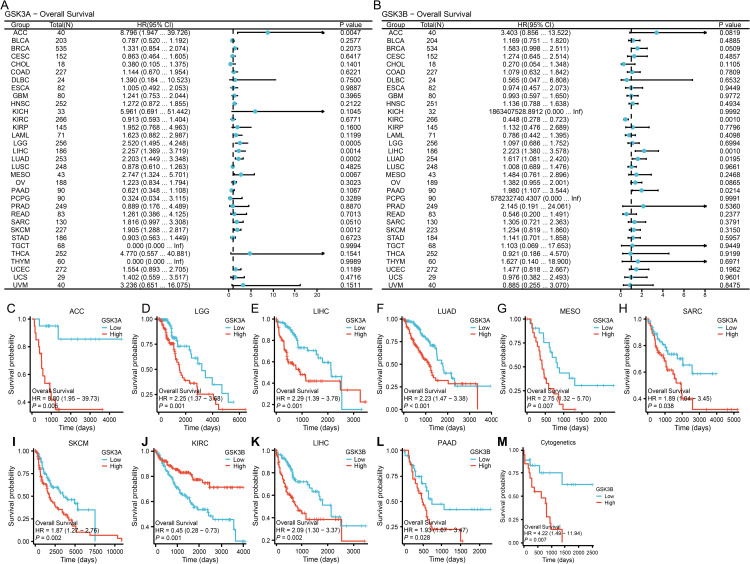
Prognostic significance of GSK3 isoforms in pan-cancer and AML subgroups. (A-B) Forest plot for the association between pan-cancer GSK3 isoforms expression and overall survival. (C-L) Kaplan-Meier curves for significant cancers. Patients with ACC (C, N = 40), LGG (D, N = 265), LIHC (E, N = 188), LUAD (F, N = 270), MESO (G, N = 44), SARC (H, N = 132), SKCM (I, N = 236), KIRC (J, N = 271), LIHC (K, N = 188), PAAD (L, N = 90). (M) Stratified AML survival: high GSK3β predicts inferior OS in cytogenetic-risk AML (N = 90). *p < 0.05, **p < 0.01, ***p < 0.001.

GSK3β emerged as an unfavorable prognostic indicator (P < 0.05, HR > 1) in LIHC (HR = 2.22, 95% CI 1.38–3.58), LUAD (HR = 1.62, 95% CI 1.08–2.42), and PAAD (HR = 1.98, 95% CI 1.11–3.54) (Fig 3B and 3K–3M-3M). Intriguingly, in KIRC (P < 0.05, HR = 0.45, 95% CI 0.28–0.72) (Fig 3J), GSK3β exhibited protective characteristics, suggesting context-dependent biological functionality. At a more profound level, we performed stratified survival analysis across cytogenetically-defined AML subgroups, revealing distinct prognostic associations of GSK3β. Patients harboring high-risk cytogenetic abnormalities – including trisomy 8 (+8), deletions 5q/del(5q) and 7q/del(7q), as well as recurrent translocations inv(16)(p13.1q22), t(15;17)(q24;q21), t(8;21)(q22;q22), and t(9;11)(p22;q23), demonstrated significantly inferior overall survival with elevated GSK3β expression (P = 0.007; HR = 4.22, 95% CI 1.49–11.94) compared to low-expression counterparts (Fig 3M).

### Immune landscape remodeling associated with GSK3α/β expression patterns

This study systematically investigated the association between GSK3α/β expression levels and immune cell infiltration within the tumor microenvironment (TME). The results revealed significant correlations between 24 cancer-related immune cell types and GSK3α/β expression. Notably, GSK3α demonstrated predominant associations with immune infiltration in ACC, TGCT, THYM, UVM. In contrast, GSK3β exhibited extensive immunomodulatory effects across most tumor types, showing particularly significant correlations with cytotoxic cells, pDC, T helper cells, Tcm and Th2 cells.

**Fig 4:**
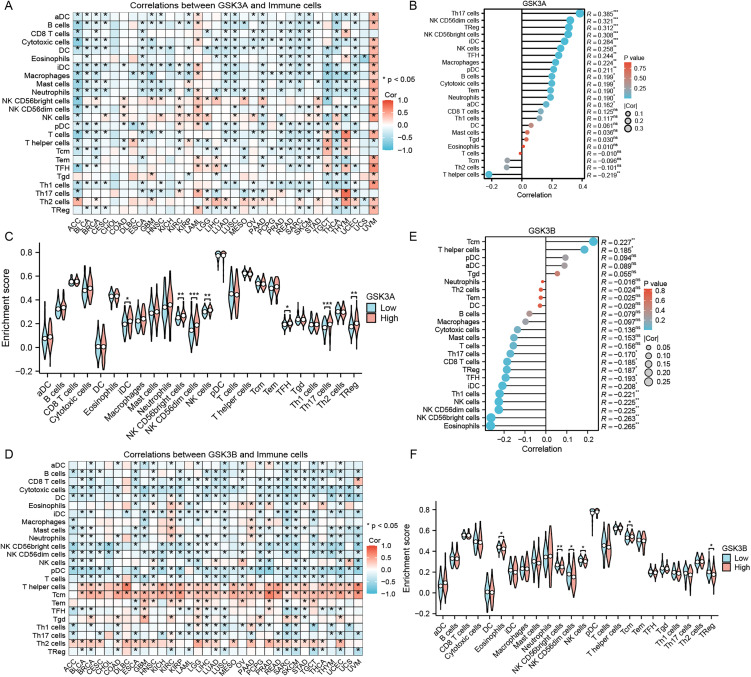
Immune microenvironment remodeling by GSK3 isoforms. (A) GSK3α expression and immune cell inﬁltration across pan-cancer. ACC: Adrenocortical carcinoma; BLCA: Bladder Urothelial Carcinoma; BRCA: Breast invasive carcinoma; CESC: cervical carcinoma; CHOL: Cholangiocarcinoma; COAD: colon adenocarcinoma; DLBC: Diffuse Large B-cell Lymphoma; ESCA: esophageal carcinoma; GBM: Glioblastoma Multiforme; HNSC: head-neck squamous carcinoma; KICH: Kidney Chromophobe; KIRC: clear cell renal cell carcinoma; KIRP: Kidney Renal Papillary Cell Carcinoma; LAML: Acute Myeloid Leukemia; LGG: lower-grade glioma; LIHC: hepatocellular carcinoma; LUAD: lung adenocarcinoma; LUSC: lung squamous carcinoma; MESO: mesothelioma; OV: ovarian cancer; PAAD: pancreatic adenocarcinoma; PCPG: Pheochromocytoma and Paraganglioma; PRAD: Prostate Adenocarcinoma; READ: Rectum Adenocarcinoma; SARC: sarcoma; SKCM: skin cutaneous melanoma; STAD: stomach adenocarcinoma; TGCT: Testicular Germ Cell Tumor; THCA: Thyroid Carcinoma; THYM: thymoma; UCEC: Uterine Corpus Endometrial Carcinoma; UCS: Uterine Carcinosarcoma; UVM: Uveal Melanoma. (B) GSK3α immunomodulation in AML. (C) Enhanced pro-tumorigenic infiltration in GSK3α-high AML. (D) GSK3β expression and immune cell inﬁltration across pan-cancer. (E) GSK3β immunosuppression in AML. (F) Reduced cytotoxic infiltration in GSK3β-high AML. *p < 0.05, **p < 0.01, ***p < 0.001.

Detailed analysis indicated moderate positive correlations between GSK3α and B cells, cytotoxic cells, mast cells, NK CD56^bright^ cells, T cells and TFH in ACC; with iDC, macrophages, and NK CD56^bright^ cells in TGCT; with T cells and Th2 cells in THYM; with eosinophils in UCS; and with natural killer cells, TFH, and Th1 cells in UVM (Fig 4A). Comparatively, GSK3β demonstrated broader immunoregulatory functions: moderate correlating with NK CD56^bright^ cells and pDC in ACC; NK CD56^bright^ cells in CHOL; T helper cells in DLBC; cytotoxic cells in GBM; pDC and Tcm in KIRP and PRAD; pDC or Tcm in PCPG, READ and UCEC; while in UVM, significant associations were observed with NK CD56^bright^ cells, natural killer cells, pDC, T helper cells and Tcm (Fig 4D). These findings suggest that GSK3β may play a more pivotal role in TME immunoregulation.

To elucidate the immunomodulatory roles of GSK3α/β in AML of tumor microenvironment, we systematically evaluated their correlation with immune cell infiltration and compared immune infiltration profiles between high- and low-expression subgroups. The analysis revealed generally weak overall correlations between GSK3α/β and immune cells in AML, but exhibited opposing regulatory patterns: GSK3α predominantly showed positive correlations with immune cells (r = 0.16–0.38, P < 0.05) (Fig 4B), whereas GSK3β demonstrated negative correlations (r = −0.17 to −0.27, P < 0.05) (Fig 4E). Notably, significant differences in immune infiltration scores were observed between expression subgroups. The GSK3α high-expression subgroup exhibited elevated enrichment levels of iDC, NK CD56^bright^, NK CD56dim, NK cells, TFH, Th17 cells and Treg (P < 0.05) (Fig 4C). Conversely, the GSK3β high-expression subgroup showed reduced infiltration of EOS, NK CD56^bright^, NK CD56dim, NK cells, Tcm and Treg (P < 0.05) (Fig 4F). These findings suggest that GSK3α and GSK3β may exert distinct immunoregulatory mechanisms within the AML microenvironment.

### Pharmacological inhibition of GSK3 exerts anti-leukemic effects in AML

**Fig 5:**
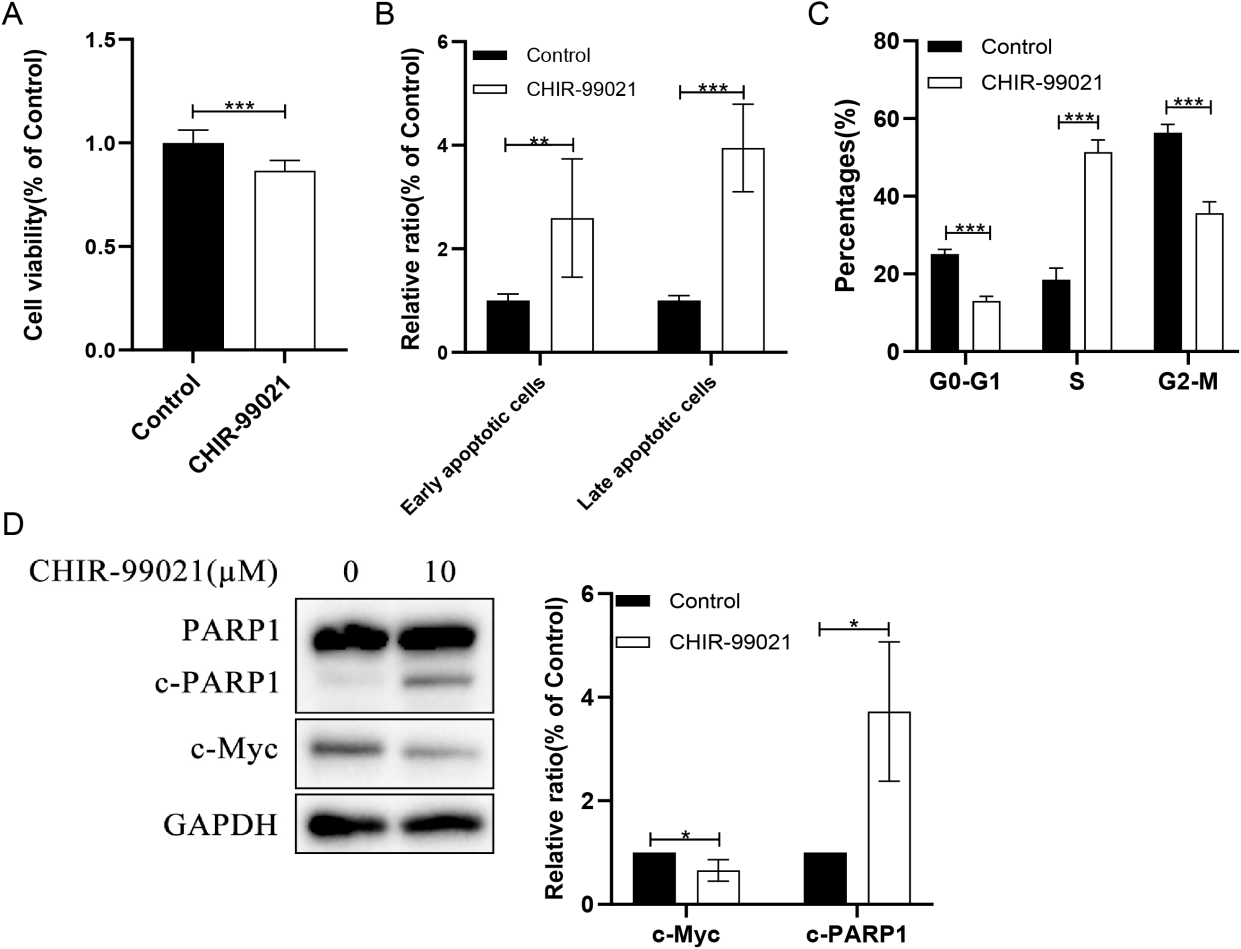
Functional validation of GSK3 inhibition in AML THP-1 cells. (A) CHIR-99021 (10 μM) inhibits cell proliferation after 48 h as measured by CCK-8 assay. 95% CI: −0.18 to −0.88, *p* < 0.0001. (B) CHIR-99021 induces apoptosis. Early apoptotic cells: 95% CI: 0.66 to 2.53, *p* = 0.005; Late apoptotic cells: 95% CI: 2.24 to 3.66, *p* < 0.0001. (C) CHIR-99021 induces S-phase arrest. S: 95% CI: 29.75 to 35.89, *p* < 0.0001. (D) Western blot analysis shows downregulation of c-Myc and accumulation of the cleaved PARP1 fragment. Data was expressed as mean ± SD for at 3 independent experiments. *p < 0.05, **p < 0.01, ***p < 0.001.

To functionally validate the role of GSK3 in AML, THP-1 cells were treated with the selective GSK3 inhibitor CHIR-99021 (10 μM). Pharmacological inhibition of GSK3 significantly suppressed cellular proliferation compared to control after 48 hours of treatment (95% CI for the difference: −0.18 to −0.88, p < 0.0001; Fig 5A). Concurrently, CHIR-99021 treatment markedly induced apoptosis, as evidenced by a significant increase in both early apoptotic (95% CI: 0.66 to 2.53, p = 0.005) and late apoptotic cell populations (95% CI: 2.24 to 3.66, p < 0.0001; Fig 5B). Cell cycle analysis revealed that this anti-proliferative effect was associated with a pronounced arrest in the S-phase (95% CI for S-phase percentage increase: 29.75 to 35.89, p < 0.0001; Fig 5C). At the molecular level, Western blot analysis confirmed functional consequences of CHIR-99021 treatment, demonstrating a clear downregulation of c-Myc (95% CI: −0.68 to −0.01, p = 0.045) along with the characteristic proteolytic cleavage of PARP1 (95% CI: 0.57 to 4.88, p = 0.025), evidenced by the accumulation of its cleaved fragment while full-length PARP1 remained unchanged (Fig 5D). Collectively, these data demonstrate that GSK3 inhibition exerts potent anti-leukemic activity in AML cells by simultaneously impairing proliferation, inducing apoptosis and cell cycle arrest, and suppressing a critical oncogenic signaling axis.

## Discussion

The present study provides a comprehensive pan-cancer analysis [24] dissecting the divergent expression patterns, clinical significance, and functional landscapes of GSK3 isoforms (GSK3α and GSK3β) across 31 malignancies, with a particular focus on acute myeloid leukemia (AML). Our integrated multi-omics analysis, combined with functional validation in AML, reveals divergent expression patterns, immune modulation, and therapeutic vulnerability. Four pivotal discoveries emerge: (1) Pan-cancer dysregulation divergence: GSK3α and GSK3β exhibit opposing expression trajectories in AML and ACC; (2) Superior diagnostic/prognostic utility of GSK3β: GSK3β outperforms GSK3α as a pan-cancer diagnostic biomarker and independently stratifies high-risk cytogenetic AML; (3) Isoform-specific immune reprogramming: GSK3α correlates with protumorigenic immune infiltration, contrasting GSK3β’s broad suppression of cytotoxic effectors in AML; (4) Pharmacological inhibition of GSK3β coordinates a concerted anti-leukemic response in AML cells, simultaneously impairing proliferation, inducing S-phase cell cycle arrest, and promoting apoptosis..

GSK3, a multifunctional kinase involved in various signaling pathways, has long been recognized for its context-dependent roles in cancer [25]. Our pan-cancer analysis revealed concurrent upregulation of both GSK3α and GSK3β in most malignancies, while notably demonstrating their coordinated downregulation in testicular germ cell tumors (TGCT). Both GSK-3 isoforms have been implicated in diverse malignancies, including colorectal cancer, breast cancer, prostate cancer, pancreatic cancer, and hematologic malignancies [26,27]. Such dysregulation corroborates the recognized function of GSK-3 [28] in promoting tumor progression via coordinated activation of Wnt/β-catenin, NF-κB, and Hedgehog signaling axes [29,30]. Paradoxically, in our results, GSK3α was found to be downregulated in both the progression of chronic myeloid leukemia (CML) and in acute myeloid leukemia (AML). However, inhibition of GSK3α has been shown to promote the differentiation of AML cells, inhibit their proliferation [31], and induce apoptosis [19]. This seemingly contradictory observation may suggest that GSK3α primarily influences disease progression in malignant hematological tumors by modulating the immune microenvironment. GSK3α functions as a double-edged sword in myeloid leukemia. Inside the AML blast, kinase activity maintains stemness and proliferation; hence its pharmacological inhibition is therapeutically beneficial. Inside cytotoxic lymphocytes [32], however, the same kinase is indispensable for metabolic fitness and anti-tumor effector functions. Global down-regulation of GSK3α in the bone-marrow micro-environment therefore simultaneously restrains AML-cell proliferation and weakens innate immune control, the net outcome depending on the relative contribution of autonomous vs. immune pressure in a given disease phase. These findings not only elucidate potential mechanisms underlying GSK3α’s role in hematopoietic malignancies but also provide novel therapeutic perspectives for targeting GSK3α. The differential expression of GSK3α and GSK3β in ACC highlights the tissue-specific oncogenic reprogramming. The upregulation of GSK3α may be closely associated with the remodeling of cellular metabolism, such as promoting glycogen synthesis to meet the energy demands of cancer cells. Meanwhile, the downregulation of GSK3β may lead to the aberrant activation of the Wnt signaling pathway, thereby promoting the proliferation and survival of tumor cells. Notably, GSK3β’s elevation in FAB M0/M1 AML—subtypes enriched for stemness and therapy resistance [33–36]—positions it as a biomarker of primitive, aggressive disease.

The prognostic and diagnostic value of GSK3α and GSK3β was further elucidated through survival analysis and ROC curve evaluation. GSK3α emerged as a significant poor prognostic factor in several cancers, including ACC, LGG, LIHC, LUAD, MESO, and SKCM, indicating its potential as a biomarker for predicting adverse outcomes. Conversely, GSK3β exhibited both unfavorable prognostic associations in LIHC, LUAD, and PAAD. GSK3β exhibited a protective association in kidney renal clear cell carcinoma (KIRC), where high GSK3β expression was correlated with better overall survival (HR = 0.45). This finding aligns with established literature demonstrating a tumor-suppressive role for GSK3β in this context; for instance, its inactivation has been shown to promote the oncogenic activities (including growth, migration, and invasion) of VHL-deficient clear cell RCC (ccRCC) in vitro and in vivo via mTOR hyperactivation [37]. Our data thus reinforce the notion that GSK3β’s functional output is highly tissue-specific, cautioning against a one-size-fits-all therapeutic approach and highlighting the need for biomarker-driven patient selection. highlighting context-dependent functionality. In terms of diagnostic capacity, GSK3β outperformed GSK3α, achieving superior AUC values in nine malignancies. GSK3β’s superior diagnostic performance likely stems from its central role in PI3K/Akt/mTOR and Wnt/β-catenin pathways frequently altered in gastrointestinal malignancies [38]. Prognostically, while GSK3α predicted poor OS in ACC and MESO—possibly through HIF1α-mediated angiogenesis [39]—GSK3β emerged as a more potent independent risk factor in cytogenetic-risk AML. This aligns with GSK3β’s ability to stabilize β-catenin and MCL1, driving survival in high-risk AML clones [31]. These results suggest that GSK3β could serve as a pan-cancer diagnostic biomarker with high discriminative power, particularly in cancers where its expression is significantly elevated.

The tumor microenvironment (TME) plays a crucial role in cancer progression and response to therapy. Our immune profiling reveals GSK3α as an immunostimulant versus GSK3β as an immunosuppressor. In AML, GSK3α-high microenvironments exhibited enriched Th17/Treg, which known to protect LSCs (leukemia stem cells) from immunosurveillance [40]. Whereas GSK3β-high microenvironments showed cytotoxic cell depletion, suggesting isoform-specific strategies for immune evasion.

The functional impact of GSK3β inhibition was validated in AML cell lines using the selective inhibitor CHIR-99021. Functional validation confirmed GSK3β’s role as an AML potential regulator. S-phase blockade with apoptosis aligns with GSK3β’s regulation of cyclin D/E-CDK complexes [41]. Given that GSK3β-high AML microenvironments are depleted of cytotoxic T and NK cells, combining GSK3β inhibitors with immune checkpoint blockade (e.g., anti-PD-1) may potentiate antitumor immunity. This is supported by recent studies showing that GSK3β inhibition enhances T cell activation and reduces Treg suppression. Clinically, GSK3β inhibitors such as lithium chloride and CHIR-99021 have been safely used in other diseases, providing a rationale for repurposing them in high-risk AML, particularly in FAB-M0/M1 subtypes with elevated GSK3β. Future studies using PDX models or immune cell co-cultures are essential to validate this hypothesis. Notably, this effect positions GSK3β inhibitors as unique agents simultaneously targeting AML vulnerabilities.

Our mechanistic investigation revealed that GSK3 inhibition in AML cells led to the downregulation of c-Myc, a key oncogenic transcription factor. This finding is significant in the context of the well-documented but context-dependent roles of GSK3β in cancer. While our data support an oncogenic role in AML, consistent with the observed pro-proliferation and anti-apoptosis phenotypes. GSK3β can function as a tumor suppressor in other malignancies, such as certain renal cell carcinomas. This duality underscores the importance of tissue-specific therapeutic strategies. Importantly, c-Myc repression is a central event in the initiation of myeloid differentiation, and its downregulation is a common consequence of treatment with differentiation-inducing agents like all-trans retinoic acid [42] (ATRA). Thus, our results provide a plausible mechanistic connection to prior studies demonstrating that GSK3 inhibition can promote differentiation in some AML models, suggesting a convergence of signaling pathways on critical nodal points like c-Myc.

Building on this connection, we propose a testable future direction: the combination of GSK3β inhibitors with established differentiation therapies. The foundational suppression of c-Myc achieved by GSK3 inhibition could potentially prime AML cells or lower their threshold for response to agents like ATRA, vitamin D analogues (e.g., 1,25-D3), or LSD1 inhibitors. Such combinations merit investigation as a strategy to overcome differentiation blockade, especially in AML subtypes poorly responsive to single agents. A limitation of our current functional study is the absence of direct surface marker analysis for terminal differentiation in the THP-1 model, which focuses the conclusions on the c-Myc-associated proliferation and survival axes. Future work should include differentiation marker assessment and explicitly test these combination therapies across a panel of AML cell lines to fully exploit the potential of GSK3β targeting in AML treatment paradigms.

Our study establishes GSK3β as a high-priority therapeutic target across multiple malignancies, particularly in AML. While establishing pan-cancer isoform landscapes, our study has some limitations. While our in vitro findings using the THP-1 cell line provide robust evidence for the anti-leukemic effects of GSK3 inhibition in an AML context, they are derived from a single cellular model. To establish broader generalizability, it would be ideal to corroborate these results across a panel of AML cell lines representing distinct genetic subtypes (e.g., HL-60, MV4−11) and, most importantly, in primary AML patient samples, particularly from high-risk groups such as FAB-M0/M1. Future studies should also explore GSK3β inhibition in co-culture systems with immune cells or in patient-derived xenograft (PDX) models to better assess its immunomodulatory impact in vivo. Second, while our functional validation demonstrates that GSK3 inhibition impairs proliferation, induces apoptosis, and arrests the cell cycle in AML cells, we were unable to assess corresponding changes in surface differentiation markers (such as CD117, CD11b, CD13) due to resource constraints following the completion of the supporting grant. The necessary antibody panels for comprehensive immunophenotyping were not available at the time of experimentation in the THP-1 model. Consequently, our analysis does not fully capture the potential differentiation-promoting effects of GSK3 inhibition. Third, bioinformatics analyses relied on bulk RNA-seq, which may mask cell-type-specific expression patterns; future studies could leverage single-cell RNA-seq to dissect TME heterogeneity.

In conclusion, this pan-cancer atlas of GSK3 isoform-specific functionality provides valuable insights into the distinct roles of GSK3α and GSK3β in cancer biology. Our findings nominate GSK3β as a master regulator and therapeutic target in AML, highlighting its potential for improving diagnostic accuracy and guiding precision medicine approaches in oncology.

## Supporting information

S1 File Original uncropped Western blot images. This file contains the original, full-length, unadjusted scans of all Western blot membranes used to generate the data panels in Figures 5D of this manuscript. Each image is clearly labeled to correspond with its respective figure panel. These raw data are also permanently available in the Zenodo repository (DOI: 10.5281/zenodo.18058235).
(PDF)

## Abbreviation:

ACC: Adrenocortical carcinoma
BLCA: Bladder Urothelial Carcinoma
CESC: cervical carcinoma
RA: rectal adenocarcinoma
OV: ovarian cancer
CRC: colorectal carcinoma
COAD: colon adenocarcinoma
ESCA: esophageal carcinoma
LIHC: hepatocellular carcinoma
LUSC: lung squamous carcinoma
SKCM: skin cutaneous melanoma
STAD: stomach adenocarcinoma
THYM: thymoma
CML: chronic myeloid leukemia
AML: acute myeloid leukemia
BC: breast carcinoma
HNSC: head-neck squamous carcinoma
PAAD: pancreatic adenocarcinoma
LGG: lower-grade glioma
LIHC: hepatocellular carcinoma
LUAD: lung adenocarcinoma
MESO: mesothelioma
SKCM: skin cutaneous melanoma
SARC: sarcoma
PAAD: pancreatic adenocarcinoma
KIRC: clear cell renal cell carcinoma
TGCT: Testicular Germ Cell Tumor
BRCA: Breast invasive carcinoma
DLBC: Diffuse Large B-cell Lymphoma
CHOL: Cholangiocarcinoma
GBM: Glioblastoma Multiforme
KICH: Kidney Chromophobe
PCPG: Pheochromocytoma and Paraganglioma
PRAD: Prostate Adenocarcinoma
READ: Rectum Adenocarcinoma
THCA: Thyroid Carcinoma
UCEC: Uterine Corpus Endometrial Carcinoma
UCS: Uterine Carcinosarcoma
UVM: Uveal Melanoma
