# Supplementary figures and images for: GSK3β as a potential regulator in AML: A pan-cancer multi-omics analysis
Source: PLoS One. 2026 Mar 31;21(3):e0344994. doi: 10.1371/journal.pone.0344994 (PMC13037974; doi:10.1371/journal.pone.0344994)

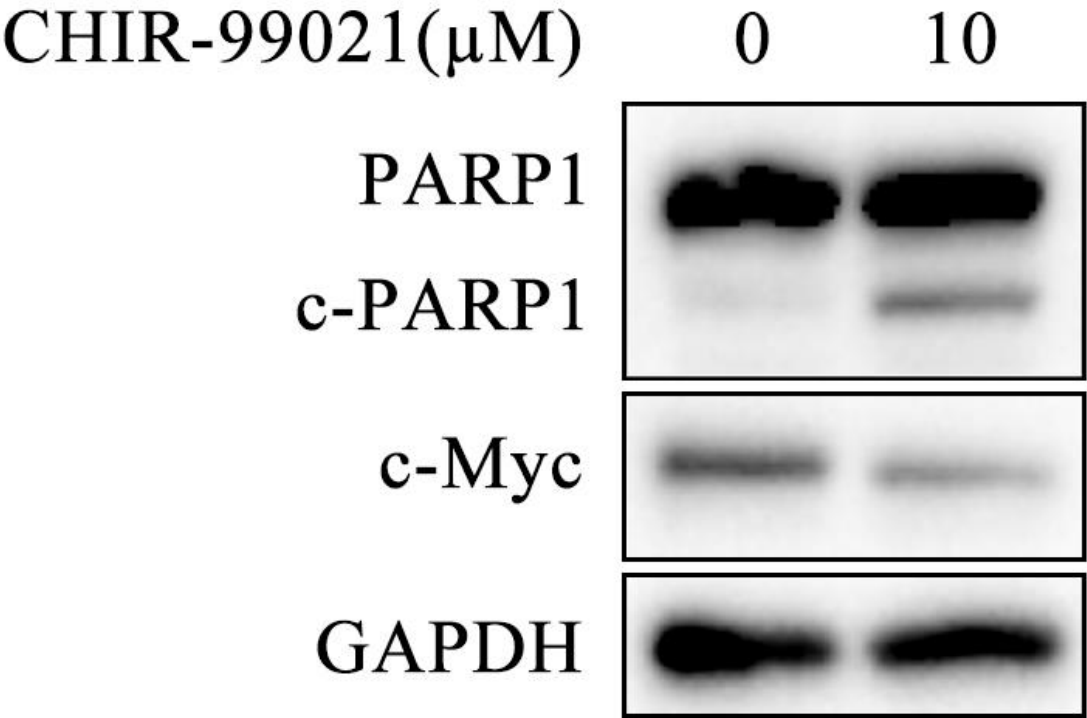

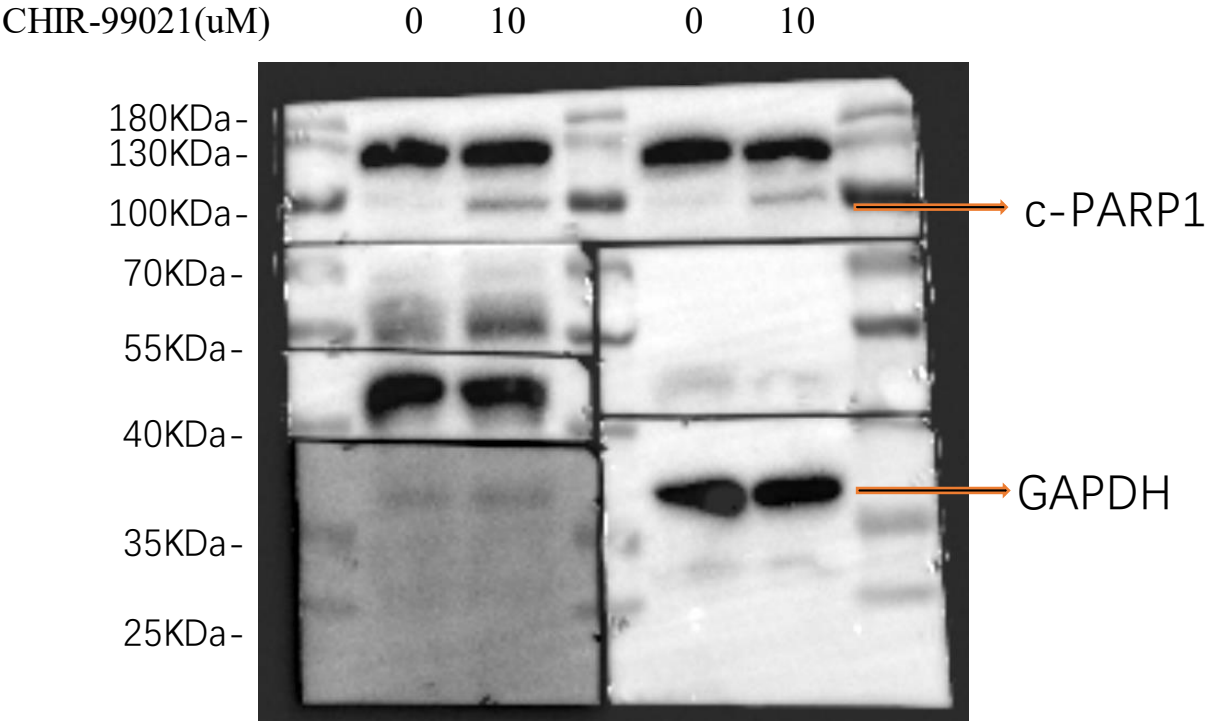

20251217-THP1 — CHIR-99021(uM)-0-10(1126)-2

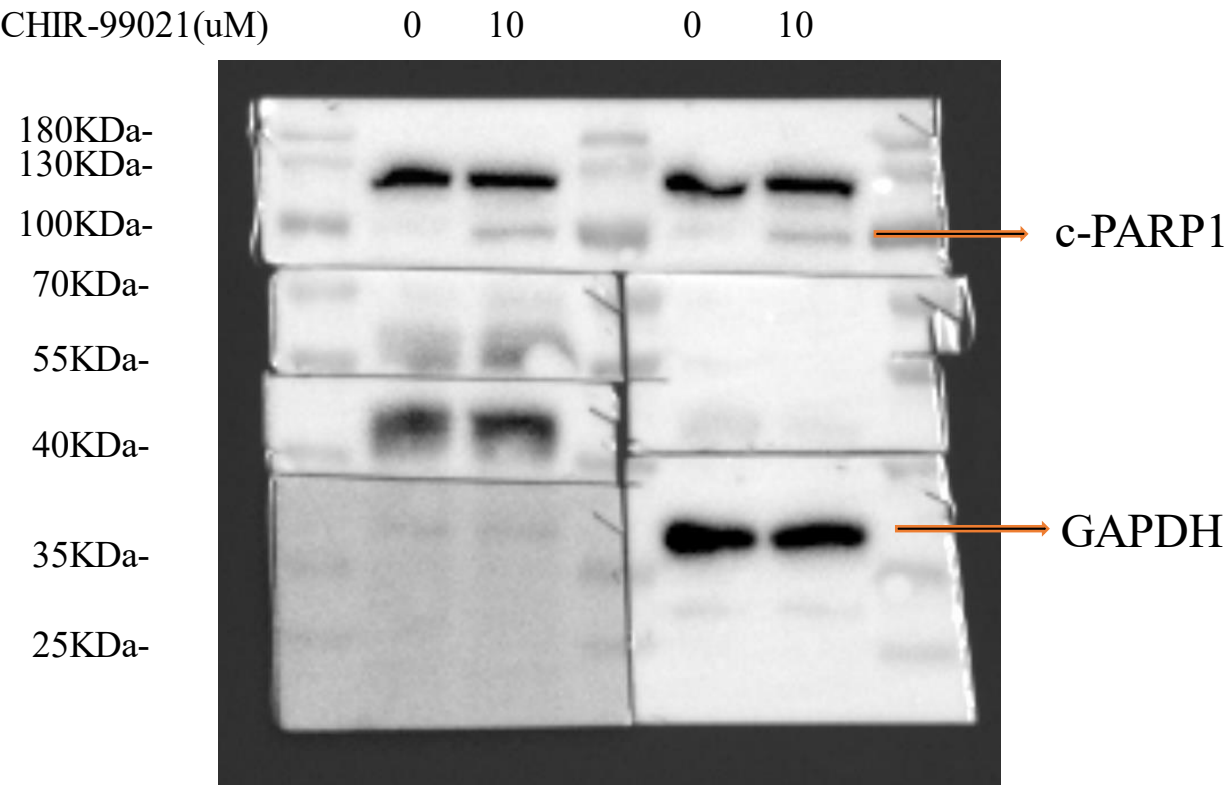

20251223-THP1 —CHIR-99021(uM)-0-10 (1126-1218)-1

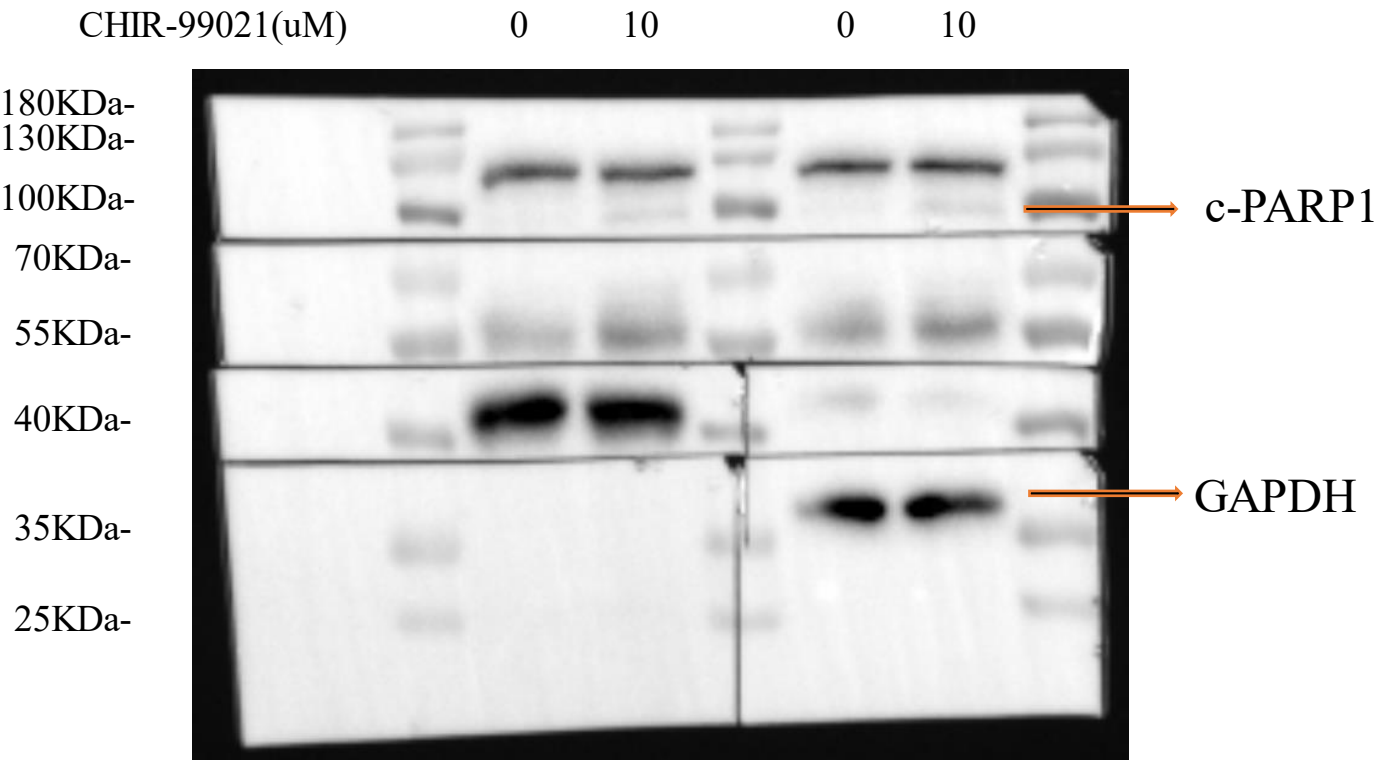

20251216-THP1 —CHIR-99021(uM)-0-10 (1210)-1

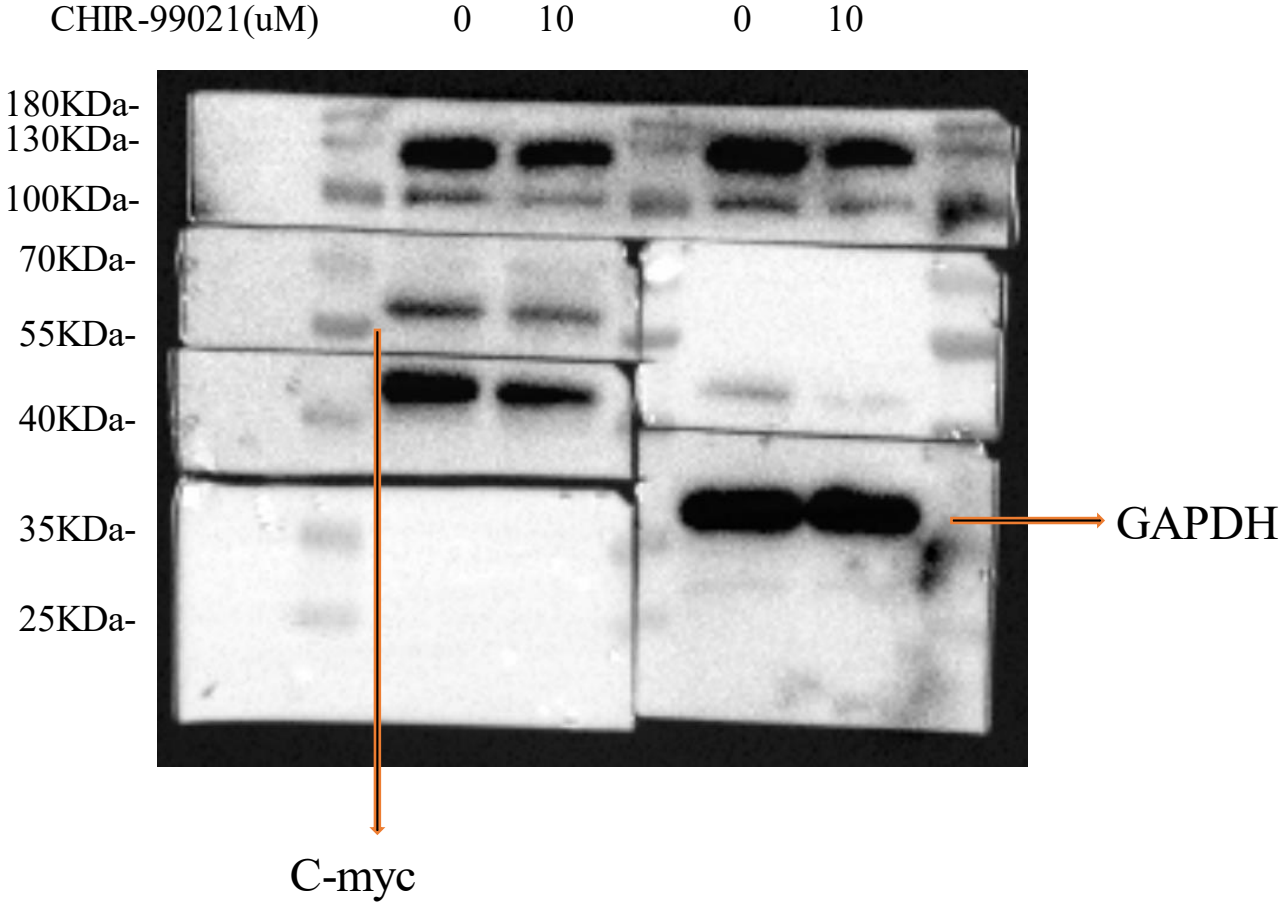

20251216-THP1 —CHIR-99021(uM)-0-10 (1210)-2

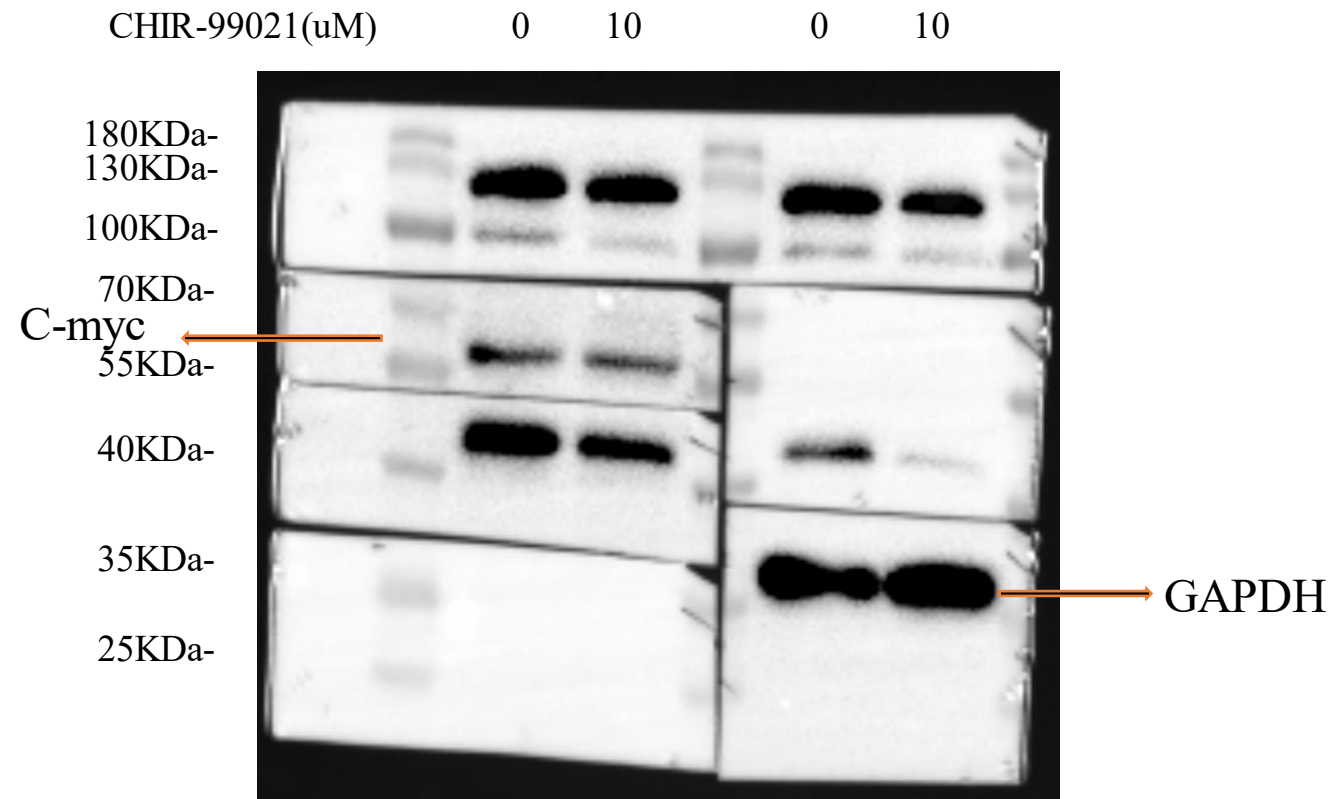

20251216-THP1- —CHIR-99021(uM)-0-10 (1210)-3

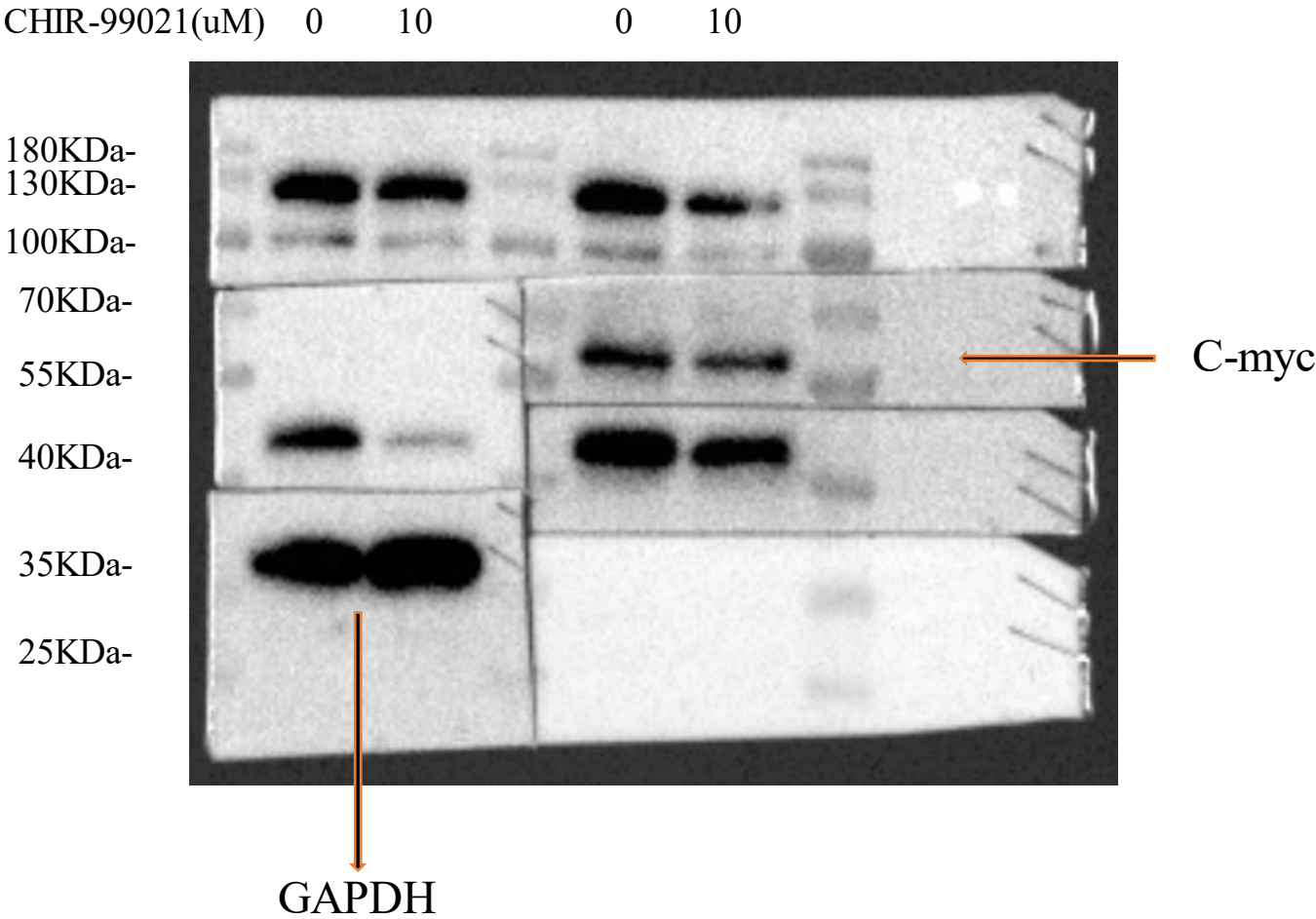

Supplement: S1 File — This file contains the original, full-length, unadjusted scans of all Western blot membranes used to generate the data panels in Figures 5D of this manuscript. Each image is clearly labeled to correspond with its respective figure panel. These raw data are also permanently available in the Zenodo repository (DOI: 10.5281/zenodo.18058235). (PDF) [file pone.0344994.s001.pdf]
